# Glass hybrid restorations as an alternative for restoring hypomineralized molars in the ART model

**DOI:** 10.1186/s12903-018-0528-0

**Published:** 2018-04-18

**Authors:** Juliana de Aguiar Grossi, Renata Nunes Cabral, Ana Paula Dias Ribeiro, Soraya Coelho Leal

**Affiliations:** 1 Faculdade de Ciências da Saúde, Universidade de Brasília, Campus Universitário Darcy Ribeiro, Asa Norte, Brasília, DF 70910-900 Brazil; 2 Department of Restorative Dental Sciences, College of Dentistry, University of Florida, 1395 Center Dr, Gainesville, FL 32610-0415 USA

**Keywords:** Molar incisor hypomineralization, Atraumatic restorative treatment, Glass ionomer, Cavity size, Survival rate

## Abstract

**Background:**

This study aimed to evaluate the survival rate of glass hybrid restorations placed under the atraumatic restorative treatment (ART) technique in first permanent molars affected by molar incisor hypomineralization (MIH).

**Methods:**

Sixty teeth with severe MIH associated to carious dentin lesions without pulp involvement were included. Treatments were performed by one trained dentist using the ART approach and restored with a glass hybrid restorative system (Equia Forte, GC®) on school premises. Treatments were evaluated after 6 and 12 months by an independent examiner using the modified ART criterion. Data analysis involved descriptive statistics and actuarial success analysis.

**Results:**

The sample comprised 24 (54.54%) girls and 20 (45.45%) boys with a mean age of 10.55 (±1.25) years. In regard to the number of surfaces involved in the restorations, 29 (48.3%) comprised one surface and 31 (51.7%) two or more surfaces. Considering cavity extent, 25 (41%) presented dentin cavitation without cusp weakness, 23 (37.7%) with large dentin cavitation with cusp weakness and 13 (21.3%) with large dentin cavitation with the breakdown of one or more cusps. Only 4 teeth required local anesthesia. A success rate of 98.3% after 6 and 12 months was observed, as only one restoration failed. The only failure occurred in a restoration involving three or more sur-faces presenting the breakdown of all cusps.

**Conclusion:**

Restorations using a glass hybrid restorative system and performed in the field with the ART technique proved, after 12 months of evaluation, to be an effective approach to preserving first permanent molars affected by MIH.

**Trial registration:**

REBEC-RBR-8drccq (17/06/15).

**Electronic supplementary material:**

The online version of this article (10.1186/s12903-018-0528-0) contains supplementary material, which is available to authorized users.

## Background

Molar incisor hypomineralization (MIH) is a relatively common condition, with its prevalence varying from 20% to 40% depending on the population studied [1, 2]. Due to its high prevalence, MIH should receive more attention, specially in developing more appropriate dental healthcare strategies to manage the problem [3]. However, it is believed that the real prevalence of MIH might not be known, as there are many methodological differences among studies carried out by different research groups [4].

MIH is defined as a developmental defect of the enamel of systemic origin that is observed in at least one of the first permanent molars, sometimes also affecting the permanent incisors [5]. Genetic variations, preterm and a number of childhood illness (such as acute otitis media, chicken pox and respiratory diseases during first years of life) are likely to be related to MIH [6]. Clinically, the lesions are characterized by demarcated opacities varying from white to a yellow/brown color surrounded by sound enamel [7]. The affected enamel is of normal thickness but of low quality in comparison with the sound enamel because of the presence of porosities that become progressively more severe as the color of the opacities changes; the darker the opacity, the lower the mineral content [6]. Often, because of masticatory forces, posteruptive breakdown of the affected enamel is observed [5, 8], which may increase sensitivity in the region due to the exposure of the dentin below the fractured enamel.

Moreover, a significant association between MIH and dental caries was found [9, 10]. Also, MIH is considered a risk factor for dental caries in populations with a low prevalence of caries [11], meaning that children who are of low caries risk have a higher chance to present the disease if affected by MIH. Chemically, the defective enamel has a high amount of carbon and a low concentration of calcium and phosphorus when compared with sound enamel [5]. Jalevik et al. (2001) [12] observed that the presence of large porosities in the microstructure of the hypomineralized enamel affects the adhesive performance of composite resin, leading to the premature loss of restorations on MIH-affected teeth. Thus, these teeth end up requiring repeated interventions, making treatment more complex and challenging [7, 13, 14].

The management of MIH is challenging as the clinical appearance and individual need for treatment varies widely [15]. Many treatment options are described for the clinical management of MIH-affected teeth that present posteruptive breakdown, such as the use of steel crowns [16], composite resin, and glass ionomer cement (GIC) [2] and also exodontia followed by orthodontia. However, consensus regarding the best restorative option for this condition is lacking in the literature [7, 8, 13, 14]. Moreover, taking into account that in low socio-economic communities, the access to dental care is limited, it is worth testing alternative treatment models for severe MIH affected teeth in which the dental equipment is not needed.

Therefore, the objective of this paper was to evaluate the survival rate of restorations performed using a new glass hybrid restorative system placed according to the atraumatic restorative treatment (ART) technique in first permanent molars affected by MIH. The choice of the ART protocol relied on the fact that it can be applied in a conventional dental setting and in field conditions [17, 18].

## Methods

### Study population

**Fig. 1:**
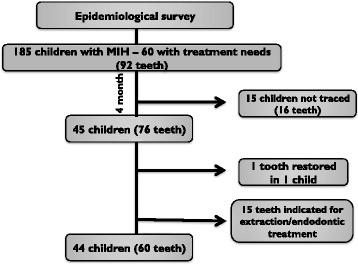
Flowchart with the number of children/eligible teeth, sample loss and composition of the study sample

The sample was composed of children aged 7 to 13 years, who were selected from a previous epidemiological survey of 1963 children from Paranoá, an underserved community located about 30 km from Brasilia, the capital of Brazil. Of these, 185 children had MIH. A survey of their treatment needs was undertaken, and those in need of restorative treatment on MIH-affected teeth were included in this clinical trial (Fig. 1). Cases in which endodontic treatment and/or dental extraction were indicated were excluded and referred to the Regional Hospital of Paranoá to receive treatment.

Parents or guardians of the selected children were interviewed by telephone regarding the socioeconomic status of the family and their knowledge of the oral health status of their children (Additional file 1).

Before the examinations were carried out, the study objectives were explained to the parents, who then signed an informed consent form. This study was approved by the Research Ethics Committee of the University of Brasilia, Brazil (CAAE-31973413.0.0000.0030) and registered as a clinical trial in the Brazilian Registry of Clinical Trials (REBEC-RBR-8drccq) with the support of the local Secretary of Education.

### Selection of cases

To determine the treatment needs of the children with MIH-affected teeth, a trained and calibrated examiner performed the clinical examinations on the school premises using portable beds, a probe, a mirror, and artificial lighting. The Nyvad criterion [19] that differentiates active from inactive carious lesions at both cavitated and non-cavitated levels, and the EAPD criterion [20] were used respectively to record dental caries and MIH. The EAPD criterion registers enamel opacities, post-eruptive breakdown, atypical restorations and tooth loss due to MIH. But in this study, it was modified as enamel breakdown with and without dentin exposure was recorded separately. Prior to the study, the examiner had been extensively calibrated on using both criteria under the supervision of an expert. Discussions and practical exercises were carried out followed by the examination of 32 children who did not participate in the main study, until an acceptable intra-examiner reliability was obtained. Kappa values of 0.95 for dental caries and 0.85 for MIH were obtained. Those teeth which presented only opacities (mild MIH) and/or opacities with posteruptive breakdown limited to the enamel (moderate MIH) were judged as not in need of invasive treatment. Teeth with posteruptive breakdown already involving dentin or those with atypical unsatisfactory restoration (severe MIH) in which there was a breakdown at the restorations margins, but that the restoration was working properly were classified as needing restoration and formed the sample of this study. MIH teeth in which pulpal exposure, fistulas, and abscesses were observed and those with major coronal destruction were indicated for endodontic treatment or exodontia. Radiographs were not used in addition to the clinical examination as all treatments were performed under field conditions. It is important to stress that the cases that were dubious, in which the examiners judged that a reliable diagnosis could not be performed without a radiograph, were excluded.

### Clinical procedures

All the restorative treatment was carried out on school premises by a single dentist and an assistant, with the child lying on a portable bed. Before beginning treatment, both the operator and the assistant were trained to perform restorations using the glass hybrid restorative system and the ART protocol [21]. The training took place at the University Hospital of Brasília, where the operator performed several restorations under the super-vision of an expert.

The carious tissue was removed with sharp excavators (Kit ART, Duflex® - Rio de Janeiro, Brazil). In some cases, when the carious tissue was involving all the hypomineralized enamel, the total affected structure was removed. Therefore, there were situations in which the restoration was placed along an MIH affected border while in others, in sound enamel. The carious removal process followed the principles of Minimum Intervention Dentistry, where the tissue was removed selectively, depending on the cavity depth [22]. In addition, unsupported enamel was removed with a hatchet specifically developed for the ART approach. If the child complained of pain, local anesthesia was administered. Once the cavity was considered ready to receive the restoration, it was classified according to the number of surfaces involved: one surface and two or more surfaces. Cavities were also classified according to the Mount & Hume classification [23] as follows: dentin cavitation without cusp weakening; extensive dentin cavitation with cusp weakening; or very extensive cavitation with destruction of one or more cusps.

The cavity was then conditioned using the Cavity Conditioner® (GC, Leuven, Belgium) for 10 s. After washing the cavity with a cotton wool pellet soaked in water, the cavity was isolated with a cotton roll, and dried with dried cotton pellets. While the operator kept the environment dry, the assistant activated the glass hybrid restorative system (Equia Forte®, GC Europe, Leuven, Belgium) in the mixer indicated by the manufacturer for 10 s. Immediately thereafter, the capsule containing the ready-to-use material was loaded into the appropriate applicator and inserted into the cavity. The material was pressed down for 40 s with a finger coated in petroleum jelly. The excess material was then removed, and the occlusion was checked using fine carbon paper. Dry cotton pellets were used to clean the surface, and the procedure was completed by applying a resinous and light-cured surface sealant (Equia Coat®, GC, Leuven, Belgium) for 20 s.

### Evaluation

One examiner (dentist) evaluated the restorations on school premises after six and 12 months using the modified ART criterion (Table 1), in which only codes 0 and 1 are considered success [24].

**Table 1:** Codes used to assess the ART restorations and their description

Code	Criteria
0	Present, stisfactory
1	Present, slight deficiency at cavity margin less than 0.5 mm
2	Present, slight deficiency at cavity margin more than 0.5 mm
3	Present, fracture in the restoration
4	Present, fracture in the tooth
5	Present, overextension of approximal margin of 0.5 mm or more
6	Not present, most or all restoration is missing
7	Not present, other restorative treatment performed
8	Not present, tooth is not present
9	Unable to diagnose

Previous to start assessing the restorations, the examiner was trained in using the criterion at paediatric clinic at University of Brasilia, where the examiner evaluated a series of ART restorations up to a kappa higher than 0.90 was obtained. Battery-illuminated dental mirrors (Kudos®, Hong Kong, China), CPITN probes, and compressed air aided in the evaluation.

### Statistical analysis

The data were analyzed using Stata Software Version 14.1. Descriptive statistics were used to present the results. The success rate of the MIH restorations was evaluated using the actuarial methods technique.

## Results

### Treatment needs

All the first permanent molars and incisors of the 185 children diagnosed with MIH during the epidemiological survey were evaluated regarding treatment needs for a total of 2200 teeth (740 M and 1480 incisors). MIH characteristics were detected in 447 M and 158 incisors (27.5%), of which 82.65% were classified as not needing invasive treatment. Of those needing treatment (105 M and 1 incisor), 5.66% of the teeth (*n* = 6) were indicated for extraction, 7.54% (*n* = 8) were referred for endodontic treatment, and 86.80% (*n* = 92) for restoration. All teeth in need of treatment had associated carious lesions.

### Characterization of lesions

Sixty teeth were restored: 31 upper molars (51.67%), 28 lower molars (46.67%) and a lower central incisor (1.67%).

Regarding MIH, all teeth were classified as severe: 57 teeth (95%) presented posteruptive breakdown with exposure of the dentin and three with (5%) unsatisfactory atypical restorations.

Considering the number of surfaces involved, 29 (48.3%) restorations involved only one surface. and 31 (51.7%) involved two or more surfaces. As for the extent of the cavity, 25 (41%) presented lesions in the dentin without compromise of the cusp, 23 (37.7%) had dentin lesions with cusp weakening, and 13 (21.3%) had dentin lesions with destruction of one or more cusps.

### Pain

Both the presence of pain and the need for local anesthesia before the beginning of treatment were recorded. Nine children reported feeling pain (15%), but only two (4 teeth) required local anesthesia. No significant associations were found between the number of surfaces involved and pain, or between the number of surfaces involved and the use of anesthesia (*p* > 0.05, Chi2).

### Success rate

**Fig. 2:**
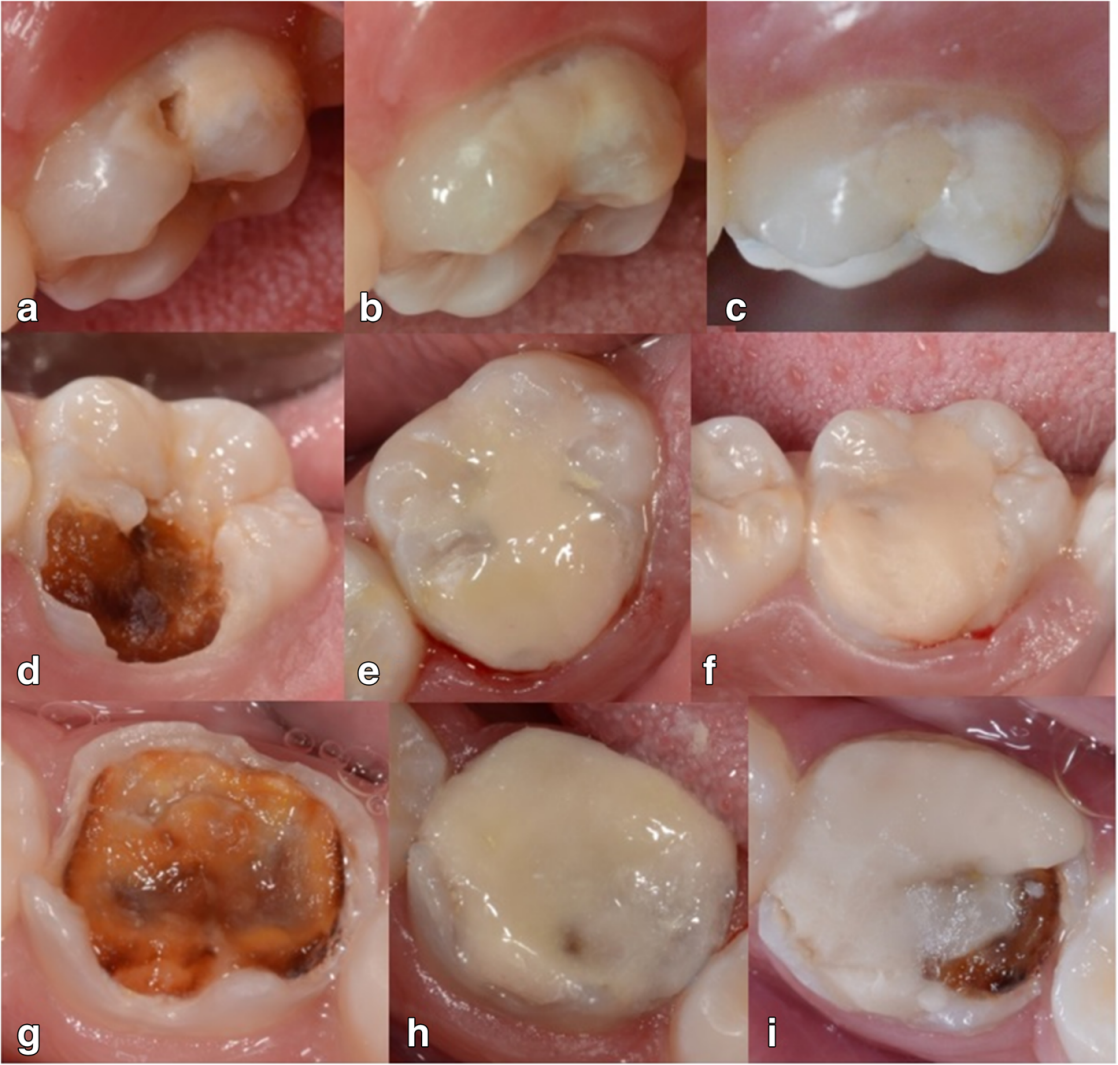
12-month follow-up and restorative failure. **a**, **d**, **g** - initial aspect of MIH affected molars associated with carious lesions at baseline; **b**, **e**, **h** - clinical aspect of restorations immediately after being performed using the ART technique involving 1 surface (**b**), 2 surfaces (**e**) and all surfaces (**h**); **c**, **f**, **i** - clinical aspect of restorations after 12 months (**c** and **d**) and the only failure observed (**i**) which occurred after 6 months follow-up

Within 6 months of evaluation, one failure was recorded (Fig. 2g-i) and was classified as 6 using the modified ART criterion [24], which characterizes fracture of the restoration and/or tooth in need of repair. The remaining restorations were classified as codes 1 (54 restorations, 90%) (Fig. 2a-f) and 2 (five restorations, 8.33%), which indicates good condition or marginal wear of less than 0.5 mm with no need for repair, respectively.

During the 6- to 12-month follow-up period, no other failure was recorded. However, four children (five restorations) were censored during this time as they were not available for evaluation. It was observed that 42 (74.78%) restorations remained classified as code 1, and 12 (22.22%) restorations as code 2. The success rate was 98.3%, as noted below in Table 2.

**Table 2:** Success of ART restorations in the 6- and 12-month periods

Interval (months)	Restorations evaluated in this period	Censored restorations	Restorations at risk during the period	Failures during the interval	Success rate during the interval	Cumulative success rate until the end of the observation period
6	60	0	60	1	0.983	0.983
12	54	5	54	0	1	0.983

### Parent/Guardian social questionnaire

The questionnaire (Additional file 1) response rate was 63.64%. The data indicated that the sample was composed of children considered to be socially vulnerable based on their family income and the level of schooling of their guardian. The income of the families did not exceed two Brazilian minimum wages (71.42%), and the guardian’s level of education, in more than half of the cases (53.57%), did not exceed eight years of study.

With regard to the oral health of the children, fewer than half of the interviewees reported being aware that their child had caries. Of those who said they knew, 53.57% said they had sought treatment, as opposed to 46.42% who had not. Of those who sought treatment, 17.85% sought private care, and 35.61% sought public service care. Of those who sought public care, only 44% were evaluated.

## Discussion

This study aimed to evaluate the success rate of restorations in MIH-affected teeth using a new restorative material and the ART protocol. The results showed a high success rate after 12 months, proving that such a strategy is a viable option for managing the problem, even without conventional dental equipment.

The present study was conducted in Paranoá, a region that has one of the lowest human development indexes in the Federal District [25]. In this context, the vast majority of families depend on the public healthcare system for dental treatment. This information is supported by the number of teeth in need of treatment and by the parents’ reports regarding the difficulties of obtaining treatment in the public service. This is not an unexpected result, since another study carried out in same region also revealed the limited access of this population to healthcare services, whether public or private [26].

Considering the vulnerability of this population, the use of the ART protocol in a school environment was, surely, the most suitable choice. ART is an approach that has been used in other countries, broadening the population’s access to healthcare services [18] and increasing the number of restorative procedures. In a study carried out in South Africa, the introduction of ART into the public service increased the number of teeth restored in both deciduous and permanent dentitions [27]**.** However, there is a lack of information about the performance of ART restorations in permanent teeth. Therefore, the restorations placed in this study must be carefully controlled, not only because of the nature of the procedure, but also due to the MIH affected tooth fragility. In this context, repair and even replacing the restorations might be needed in the future. In addition, the reduced need for local anesthesia in the ART approach is an advantage, especially when unaccompanied children are being treated. ART has proved to be especially important in the behavior management of children when the dentist is not pediatric specialist [17], as was the case in the present investigation. Of the 44 children treated, only two (four teeth) required local anesthesia during the procedure. The low need for local anesthesia related to ART is probably due to the exclusive use of hand instruments to access and clean the cavity, but this is a hypothesis that calls for further investigation.

The success of restorations after 12 months of follow-up was 98%, considered a high success rate. This result was better than that found by Fragelli et al. (2014) [2], in which a 78% success rate was achieved in restorations performed on teeth affected by MIH. The difference with respect to survival rates between the present investigation and the study performed by Fragelli et al., (2014) [2] may be due to the restorative technique and the type of restorative material used. In the present investigation, the restorations were placed using the ART technique, in which absolute isolation is not used, and a new encapsulated hybrid restorative system. Fragelli et al. (2014) [2] used a high viscosity glass ionomer cement that was hand mixed.

Regarding the type of material, this is the first clinical study to Equia Forte® (GC, Leuven, Belgium) for severely affected MIH teeth, making it impossible to compare these results with similar studies. However, Gurgan et al. (2015) [28] evaluated the clinical performance of Equia Fil® (GC, Leuven, Belgium) with a surface protector (Equia Coat®, GC, Leuven, Belgium), precursor of the Equia Forte®, for managing carious lesions in premolars and permanent molars for four years in comparison with the microhybrid composite resin. The authors showed that the clinical performance of Equia Fil® was similar to that of composite resin for both class I and class II restorations [28]. One of the differentials of this hybrid restorative system is the surface protection of the restorations, which is accomplished by applying a light-cure resin sealant that seems to improve the final smoothness of the restoration and reduce surface wear. The material used in the present study seems to have had a positive influence on the survival outcomes.

Composite resin was recently tested as a restorative material for MIH teeth in a clinical trial. After 12 months of control, the survival rate of the restorations performed with the self-etching adhesive system was 73% and 59% with the total-etching adhesive system [29]. Comparing the success rate obtained in the present study (98%) in the same period, better behavior of the hybrid system tested here was noted. These results can be partly explained by inherent differences in the sample, but also by the poor adhesion already observed between composite resin and enamel edges affected by MIH [13, 15]. The formation of tags after acid etching—an extremely important step in resin adhesion— is deficient and negatively affects the retention of the restoration [13, 29, 30]. Hence, the removal of all opacity [31, 32] has been recommended to allow the adhesion of the resin to sound enamel. However, this is an extremely invasive strategy, especially for those cases in which the entire crown of the tooth is affected and where the child is quite young**.** In the present study, opacities were only removed when there was carious tissue present or were located in unsupported enamel. Another management option would be steel crowns, which are widely used and recommended in the United States and Europe [16, 31]. However, these were not considered in this study, since they are not available on the Brazilian market.

As only one failure was detected during the observational period, more robust statistical analysis such as Kaplan Meier regression and Cox regression could not be applied. Initially, this aspect can be seen as a limitation, but it does stress the excellent results obtained in the present investigation. An observational analysis of the factors that influenced the success of the treatment showed that the only failure occurred in a cavity in which all surfaces were involved and which was already presenting the destruction of all cusps (Fig. 2g,). Because of this, we infer that any other type of restorative material placed with the direct technique would have had a greater chance of failure. Although direct restoration may be contraindicated in such a case, it was done because the child’s mother could not afford private treatment and did not have time to take her child to the local hospital for free treatment. In general, although the results have been very promising, other studies using the glass hybrid restorative system should confirm these data before recommending the system as the material of choice for direct restorations in teeth affected by MIH in young children, even in areas of masticatory effort.

This study presents has limitations. They include reduction in the initial sample, sample selection, and absence of a control group. Initially, the study identified 185 children with MIH, of whom 60 (92 teeth) had restorative needs. However, the implementation of the present investigation was only achieved 4 months after the epidemiological survey had been completed, and after the children had returned from their summer vacation, reducing the sample number to 45 children. Of these children, who initially presented 76 teeth with MIH and restoration needs, only 60 teeth could be treated (Fig. 1). This happened since 15 teeth were assessed at the moment of the intervention as in need of endodontics/exodontia, demonstrating the rapid destruction of teeth affected by MIH because of enamel porosity in association with carious lesions. Such results are supported by the literature and demonstrate the need for the early diagnosis of MIH. When restorative needs are already present, treatment must be performed as soon as possible [7, 30, 32, 33]. When not treated immediately, more complex therapies and greater costs are entailed, making the problem even more difficult to solve. Moreover, the power of the study can be questioned because of the number of restorations performed. However, the present investigation was able to include a greater number of affected teeth (60) in comparison with studies with a similar aim: 48 and 41 teeth respectively [2, 29]. This shows the difficulties of working with very specific conditions such as MIH.

Another limitation is the lack of a control group. This is justified by the fact that no consensus protocol exists regarding the treatment of MIH teeth [2, 7, 8, 16, 30, 31], which would indicate the best material/technique to be used as a comparison. Two systematic reviews that aimed to assess the modalities of treatments available to manage affected MIH teeth concluded that that there is a lack of information provided by long-term clinical trials with respect to the management of the condition, not allowing strong recommendations to be made with respect to the best protocol [14, 15].

## Conclusion

The majority of MIH teeth did not need invasive treatment, and those that did need treatment required mostly direct restorations. Restoration using a glass hybrid restorative system and performed in the field with the ART technique proved to be an effective approach to preserving first permanent molars affected by MIH.

## Additional file

Additional file 1: Parent/guardian social questionnaire: parents or guardians of the selected children were interviewed by telephone regarding the socioeconomic status of the family and their knowledge of the oral health status of their children. (PDF 82 kb)

## Abbreviations

ART: Atraumatic restorative treatment
DF: Federal District
EAPD: European Academy of Paediatric Dentistry
GIC: Glass ionomer cement
MIH: Molar incisor hypomineralization
SUS: Brazilian Public Health System

## References

[1] Soviero V, Haubek D, Trindade C, Da Matta T, Poulsen S (2009). Prevalence and dis-tribution of demarcated opacities and their sequelae in permanent 1st molars and incisors in 7 to 13 year-old Brazilian children. Acta Odontol Scand.

[2] Fragelli CMB, Souza JF, Jeremias F, Cordeiro RCL, Santos-Pinto L (2015). Molar incisor hypomineralization (MIH): conservative treatment management to restore affected teeth. Braz Oral Res.

[3] Zhao D, Dong B, Yu D, Ren Q, Sun Y. The prevalence of molar incisor hypomineralization: evidence from 70 studies. Int J Paediatr Dent. 2017; 10.1111/ipd.12323. 10.1111/ipd.12323 28732120

[4] Hernandez M, Boj JR, Espasa E (2016). Do we really know the prevalence of MIH?. J Clin Pediatr Dent.

[5] Weerheijm KL, Jalevik B, Alaluusua S (2001). Molar incisor hypomineralization. Caries Res.

[6] Chawla N, Messer LB, Silva M (2008). Clinical studies on molar incisor Hypomineralisation part 1: distribution and putative associations. Eur Arch Paediatr Dent.

[7] Takahashi K, Correia ASC, Cunha RF (2009). Molar incisor hypomineralization. J Clin Pediat Dent.

[8] Lygidakis NA, Wong F, Vierrou AM, Alaluusua S, Espelid I (2010). Best clinical practice guidance for clinicians dealing with children presenting with molar incisors hypomineralisation (MIH). An EADP policy document. Eur Arch Paediatr Dent.

[9] Grossi JA, Cabral RN, Leal SC (2017). Caries experience in children with and without molar-incisor Hypomineralisation: a case-control study. Caries Res.

[10] Americano GC, Jacobsen PE, Soviero VM (2017). Haubek D.- a systematic review on the association between molar incisor hypomineralization and dental caries. Int J Paediatr Dent.

[11] Garcia-Margarit M, Catalá-Pizarro M, Montiel-Company JM, Almerich-Silla JM (2014). Epidemiologic study of molar-incisor hypomineralization in 8-year-old Spanish children. Int J Paediatr Dent.

[12] Jälevik B, Norén JG (2000). Enamel hypomineralization of permanent first molars: a morphological study and survey of possible aetiological factors. Int J Paediatr Dent.

[13] Jälevik B, Klingberg GA (2002). Dental treatment, dental fear and behaviour management problems in children with severe enamel hypomineralisation of their permanent first molars. Int J Paediatr Dent.

[14] Lygidakis NA (2010). Treatment modalities in children with teeth affected by molar-incisor enamel hypomineralisation (MIH): a systematic review. Eur Arch Paediatr Dent.

[15] Elhennawy K, Schwendicke F (2016). Managing molar-incisor hypomineralization: a systematic review. J Dent.

[16] Koch MJ, García-Godoy F (2000). The clinical performance of laboratory fabricated crowns placed on first permanent molars with developmental defects. JADA.

[17] Frencken JE, Leal SC, Navarro MFL (2012). 25 years atraumatic restorative treatment (ART) approach: a comprehensive overview. Clin Oral Invest.

[18] Luengas-Quintero E, Frencken JE, Muñúzuri-Hernández JA, Mulder J (2013). The atraumatic restorative treatment (ART) strategy in Mexico: two-years follow up of ART sealants and restorations. BMC Oral Health.

[19] Nyvad B, Machiulskiene V, Baelum V (1999). Reliability of a new caries diagnostic system differentiating between active and inactive caries lesions. Caries Res.

[20] Weerheijm KL, Duggal M, Mejare I, Papagiannoulis L, Koch G, Martens LC, Hallonsten AL (2003). Judgement criteria for molar incisor hypomineralization (MIH) in epidemiologic studies: a summary of European meeting on MIH held in Athens, 2003. Eur J Paed Dent.

[21] Frencken JE, Holmgren CJ (1999). Atraumatic restorative treatment for dental caries.

[22] Schwendicke F, Frencken JE, Bjørndal L, Maltz M, Manton DJ, Ricketts D, Van Landuyt K, Banerjee A, Campus G, Doméjean S, Fontana M, Leal S, Lo E, Ma-chiulskiene V, Schulte A, Splieth C, Zandona AF, Innes NP (2016). Managing carious lesions: consensus recommendations on carious tissue removal. Adv Dent Res.

[23] Lasfargues JJ, Kaleka R, Louis JJ. A new system of minimally invasive preparations: The Si/Sta concept. In: Roulet JF & Degrange M Adhesion: The Silent Revolution in Dentistry. Chicago: Quintessence; 2000. p. 107–151.

[24] Farag AM, Van der Sanden WJ, Abdelwahab H, Frencken JE (2011). Survival of ART restorations assessed using selected FDI and modified ART restoration criteria. Clin Oral Investig.

[25] Atlas de Desenvolvimento Humano no Brasil. 2013. http://www.atlasbrasil.org.br/2013/pt/perfil_udh/22939. Accessed 20 Sept 2016.

[26] De Amorim RG, Figueiredo MJ, Leal SC, Mulder J, Frencken JE (2012). Caries experience in a child population in a deprived area of Brazil, using ICDAS II. Clin Oral Investig.

[27] Mickenautsch S, Frencken JE (2009). Utilization of the ART approach in a group of public oral health operators in South Africa: a 5-year longitudinal study. BMC Oral Health.

[28] Gurgan S, Kutuk ZB, Ergin E, Oztas SS, Cakir FY (2015). Four-year randomized clinical trial to evaluate the clinical performance of a glass ionomer restorative system. Oper Dent.

[29] De Souza JF, Fragelli CB, Jeremias F, Paschoal MAB, Santos-Pinto L, Cordeiro RCL. Eighteen-month clinical performance of composite resin restorations with two different adhesive systems for molars affected by molar incisor hypomineralization. Clin Oral Invest. 2016; 10.1007/s00784-016-1968-z. 10.1007/s00784-016-1968-z 27743215

[30] Garg N, Jain AK, Saha S, Singh J (2012). Essentiality of early diagnosis of molar incisor Hypomineralization in children and review of its clinical presentation, etiology and management. Int J Clin Pediatr Dent.

[31] William V, Messer LB, Burrow MF (2006). Molar incisor Hypomineralisation: review and recommendations for clinical management. Pediatr Dent.

[32] Fayle SA (2003). Molar-incisor hypomineralisation: restorative management. Eur J Paediatr Dent.

[33] Preusser SE, Ferring WC, Wetzel WE (2007). Prevalence and severity of molar incisor hypomineralization in a region of Germany - a brief communi-cation. J Public Health Dent.

