# Supplementary material for: Glass hybrid restorations as an alternative for restoring hypomineralized molars in the ART model
Source: BMC Oral Health. 2018 Apr 18;18:65. doi: 10.1186/s12903-018-0528-0 (PMC5907471; doi:10.1186/s12903-018-0528-0)
Supplement: Supplementary file 1 — Parent/guardian social questionnaire: parents or guardians of the selected children were interviewed by telephone regarding the socioeconomic status of the family and their knowledge of the oral health status of their children. (PDF 82 kb) [file 12903_2018_528_MOESM1_ESM.pdf]

PARENT/GUARDIAN SOCIAL QUESTIONNAIRE

Name of the respondent:____

Father ( ) Mother ( ) Other ( )

Telephone:____

Child`s name/ID: ____

Information about the parent who is replying this questionnaire

1. Do you have a job?? ☐ Yes ☐ No

   Profession:________________________________.

2. Residence: house ☐ apartment ☐

   ☐ Own house ☐ rented ☐ other:__________________

3. How many people live with you?______.
4. How many people have a job?______.
5. Monthly family income:

☐ at least 1 brazilian minimum wages (BMW) ☐ more than de 1 until 2 BMW
☐ more than de 2 until 5 BMW ☐ more than 5 until 8 BMW
☐ more than 8 BMW

6.Did you attend any school? ☐ Yes ☐ No

☐ elementary school ☐ secondary school
☐ high school ☐ college education

7. Did you know that your child had dental caries? ☐ Yes ☐ No
8. If yes, did you know it was permanent tooth? ☐ Yes ☐ No
9. If yes, did you seek for any dental treatment to solve the problem?

   ☐ Yes ☐ No

10. If yes, was it private clinic or a public oral health center?

    ____

11. If in a public oral health center, were you able to make an appointment?

    ☐ Yes ☐ No
